# Understanding the impact of fibroblast heterogeneity on skin fibrosis

**DOI:** 10.1242/dmm.044164

**Published:** 2020-06-17

**Authors:** Michelle F. Griffin, Heather E. desJardins-Park, Shamik Mascharak, Mimi R. Borrelli, Michael T. Longaker

**Affiliations:** 1 Hagey Laboratory for Pediatric Regenerative Medicine, Division of Plastic Surgery, Department of Surgery, Stanford, CA 94305, USA; 2 Stanford Institute for Stem Cell Biology and Regenerative Medicine, Stanford University School of Medicine, Stanford, CA 94305, USA

**Keywords:** Fibroblast heterogeneity, Scarring, Wound healing, Dermis, Skin fibrosis

## Abstract

Tissue fibrosis is the deposition of excessive extracellular matrix and can occur as part of the body's natural wound healing process upon injury, or as a consequence of diseases such as systemic sclerosis. Skin fibrosis contributes to significant morbidity due to the prevalence of injuries resulting from trauma and burn. Fibroblasts, the principal cells of the dermis, synthesize extracellular matrix to maintain the skin during homeostasis and also play a pivotal role in all stages of wound healing. Although it was previously believed that fibroblasts are homogeneous and mostly quiescent cells, it has become increasingly recognized that numerous fibroblast subtypes with unique functions and morphologies exist. This Review provides an overview of fibroblast heterogeneity in the mammalian dermis. We explain how fibroblast identity relates to their developmental origin, anatomical site and precise location within the skin tissue architecture in both human and mouse dermis. We discuss current evidence for the varied functionality of fibroblasts within the dermis and the relationships between fibroblast subtypes, and explain the current understanding of how fibroblast subpopulations may be controlled through transcriptional regulatory networks and paracrine communications. We consider how fibroblast heterogeneity can influence wound healing and fibrosis, and how insight into fibroblast heterogeneity could lead to novel therapeutic developments and targets for skin fibrosis. Finally, we contemplate how future studies should be shaped to implement knowledge of fibroblast heterogeneity into clinical practice in order to lessen the burden of skin fibrosis.

## Abstract (precis)

**Summary:** This Review discusses the multifaceted aspects of fibroblast heterogeneity and the different roles of fibroblast subpopulations to help overcome skin scarring and fibrosis.

## Introduction

Fibrosis is the replacement of functional connective tissue with excessive collagen-rich extracellular matrix (ECM). This results in the formation of fibrotic scars, which are the inevitable consequence of the body's repair process following tissue damage (Coentro et al., 2018). Fibrosis is characterized by fibroblast proliferation and deposition of excessive pathological ECM, and can affect any organ, leading to progressive tissue scarring and organ dysfunction. When all causes of fibrosis (including acute injury, chronic degeneration) are considered, organ fibrosis is estimated to contribute to nearly 50% of all deaths in the developed world (Friedman et al., 2013). Skin fibrosis can manifest locally in response to dermal injury following burn, surgery, trauma, infection or radiation, or in association with systemic diseases such as scleroderma and graft-versus-host disease (Pedroza et al., 2018; Song et al., 2018). When skin fibrosis becomes excessive, hypertrophic scars or keloids form.

The global impact of skin fibrosis is significant, with over 100 million people affected every year in the developed world (Bayat et al., 2003). Scarring can detrimentally affect patients' quality of life due to the cosmetic disfigurement and consequent psychosocial distress (Bock et al., 2006). Despite the expansive market for anti-scarring medication, estimated to be in excess of $12 billion per year in the United States, no universal effective anti-scarring treatment exists (Jiang et al., 2018). Thus, the ability to repair cutaneous injuries without fibrosis would reform clinical practice and avoid the significant morbidity associated with lacerations, surgical incisions and burns.

Skin repair involves restoring tissue integrity through a complex and tightly controlled process that consists of the following overlapping stages: homeostasis, inflammation, proliferation and maturation (Eming et al., 2014). Several cell types, cytokines and growth factors involved in specific signaling pathways cooperate and coordinate to execute these steps (Cañedo-Dorantes and Cañedo-Ayala, 2019). Fibroblasts are central to all stages of wound healing and those found in the skin are the most abundant mesenchymal cell in the dermis (Li and Wang, 2011). For a long time, it was assumed that fibroblasts were a homogenous, static population of spindle-shaped cells (Ravikanth et al., 2011). However, emerging evidence indicates that fibroblasts are actually a morphologically and functionally heterogeneous cell population. This has led to a fresh perspective on dermal fibrosis, specifically on the critical role that fibroblast heterogeneity plays, not only in skin homeostasis but also in pathology, such as scarring and fibrosis (Sriram et al., 2015).

This Review aims to discuss the current knowledge of the role of fibroblast heterogeneity in wound healing and fibrosis. We highlight the differences in fibroblast heterogeneity observed in human and mouse dermis, and summarize current insight into the understanding of cell-cell and cell-matrix communications that may shape fibroblast heterogeneity. Lastly, we contemplate how fibroblast heterogeneity could lead to the development of more-effective therapeutic modalities for wound healing.

## Skin architecture and the role of fibroblasts

**Fig. 1.**
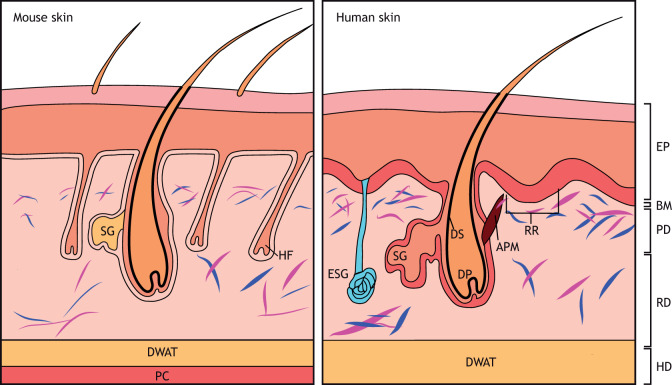
**Schematic illustration of the skin in mouse and humans.** Left: murine skin structure. Mouse skin has a high density of fibroblasts (blue and purple). The panniculus carnosus is under the hypodermis. Right: human skin structure. Human skin structure differs from that of mouse. The epidermis is thicker and forms ingrowths called rete ridges (RR). Hair follicle density in human skin is lower than in mouse. APM, arrector pili muscle; BM, basement membrane; DP, dermal papillae; DS, dermal sheath; DWAT, dermal white adipose tissue; EP, epidermis; ESG, eccrine sweat gland; HD, hypodermis; HF, hair follicle; PC, panniculus carnosus; PD, papillary dermis; RD, reticular dermis; SG, sebaceous gland. Adapted with permission from Rippa et al. (2019). This image is not published under the terms of the CC-BY license of this article. For permission to reuse, please see Rippa et al. (2019).

Skin is composed of two organized layers: a more superficial epidermal layer, mainly composed of keratinocytes, and a deeper dermal layer, primarily consisting of fibroblasts (Schoop et al., 1999). There are many parallels between mouse and human skin (Fig. 1) (Rippa et al., 2019; Sriram et al., 2015). In both, fibroblasts are the most abundant cell type in the dermis and are responsible for laying down the ECM (Rinkevich et al., 2015). Traditionally, fibroblasts are defined by their spindle-shaped morphology (Mollenhauer and Bayreuther, 1986), adhesion to tissue culture plastic (Rusnati et al., 1997), expression of known mesenchymal markers including collagen I and vimentin, and lack of expression of specific cell lineage markers, such as endothelial, epithelial or immune markers (Kokkinos et al., 2007; Sriram et al., 2015). Notably, despite the historically simple description of fibroblast phenotype, different fibroblasts exhibit distinct gene expression patterns and different functions (Philippeos et al., 2018). The murine and human unwounded dermis exhibits functional fibroblast diversity on several levels, as dictated by (1) their embryonic origin, (2) the tissue anatomical site, and (3) the microenvironment and localization within the tissue (Lynch and Watt, 2018; Sriram et al., 2015).

## Examining fibroblast heterogeneity in human and mouse skin

**Fig. 2.**
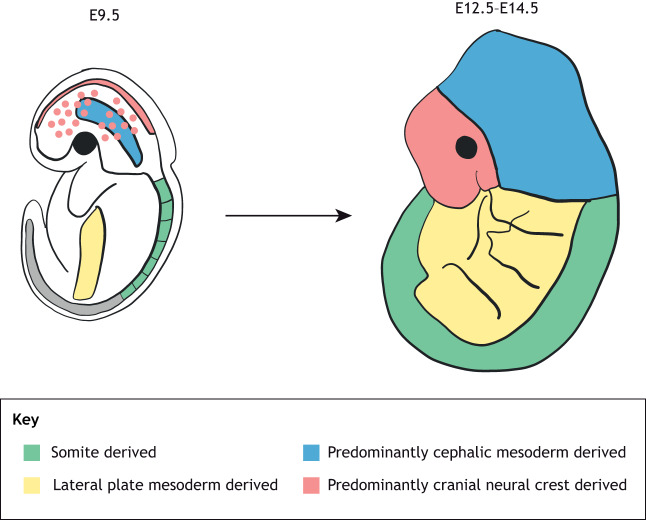
**Schematic to show the embryonic origin of dermal fibroblasts.** The origins of the dermis from different sites of the body are different. The dorsum dermis originates primarily from the somite, the ventrum dermis from the lateral plate, the cranial dermis from the cephalic mesoderm and the face dermis from the neural crest. E, embryonic day. Adapted with permission from Thulabandu et al. (2018). This image is not published under the terms of the CC-BY license of this article. For permission to reuse, please see Thulabandu et al. (2018).

Cell fate-mapping experiments have demonstrated that the differences in the embryological origin of dermal fibroblasts in human and mice depend on the anatomical location of the body (Jiang et al., 2002; Ohtola et al., 2008). Fibroblasts residing in the skin of the face are derived from the neural crest (see Glossary, Box 1), whereas those located within the dorsum originate from the dermato-myotome (see Glossary, Box 1) and those within the ventral dermis are derived from the lateral plate of the mesoderm (see Glossary, Box 1) (Thulabandu et al., 2018; Wong et al., 2006; Yamaguchi et al., 1999) (Fig. 2). To analyze fibroblast heterogeneity in the skin due to their embryonic origin, it is important to have markers that define fibroblast cell subpopulations.

**Box 1. Glossary**

**Dermato-myotome:** the origin of the dorsal dermis.

**HOX code:** describes a number of rules regarding the expression of homeobox (HOX) genes and their effects on segment identity.

**Mesoderm:** one of the three primary germ layers that are sandwiched between the two other germ layers known as ectoderm and endoderm.

**Myofibroblast:** differentiated fibroblasts that express α-SMA.

**Neural crest:** a transient embryonic structure in vertebrates that gives rise to the peripheral nervous system and to several non-neural cell types.

**Stroma:** the supportive tissue of an epithelial origin.

**Wound contraction:** healing response following skin wounding that reduces the size of the tissue defect to decrease the amount of tissue that needs repair.

**Fig. 3.**
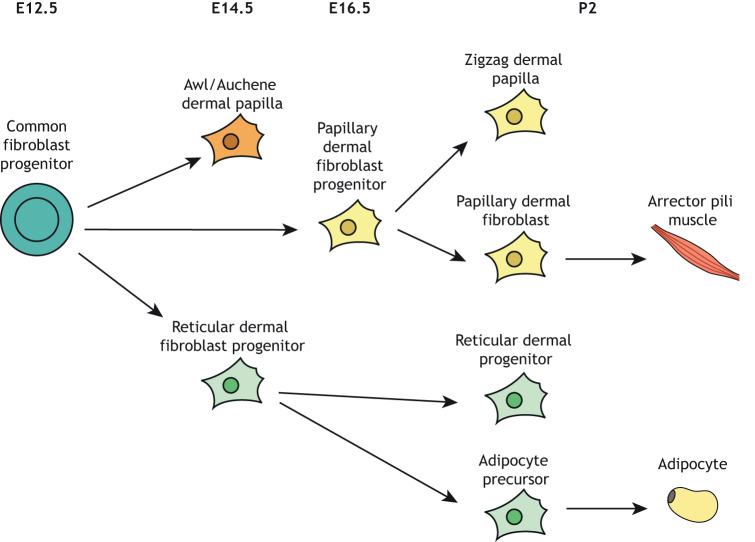
**Schematic of the dermal fibroblast lineages.** All fibroblasts originate from a common fibroblast progenitor. In mouse, this process starts at E12.5. Papillary dermal fibroblast progenitors give rise to zig-zag dermal papilla and papillary dermal fibroblasts. Reticular dermal fibroblast progenitors give rise to reticular dermal fibroblasts and adipocytes. E, embryonic day; P, postnatal day. Adapted with permission from Driskell et al. (2013). This image is not published under the terms of the CC-BY license of this article. For permission to reuse, please see Driskell et al. (2013).

Several putative ‘pan-fibroblast’ markers have been well studied in the mouse, including platelet-derived growth factor receptor alpha (PDGFRA) (Philippeos et al., 2018). Using lineage tracing, Driskell et al. (2013) demonstrated that mouse dermal fibroblasts arise from a multipotent progenitor population that expresses PDGFRA, delta-like non-canonical Notch ligand 1 (DLK1) and leucine-rich repeats and immunoglobulin-like domains protein 1 (LRIG1) (Driskell et al., 2013) (Table 1). This population has the capacity to differentiate into all dermal fibroblast lineages (Driskell et al., 2013) (Fig. 3).

**Table 1.**
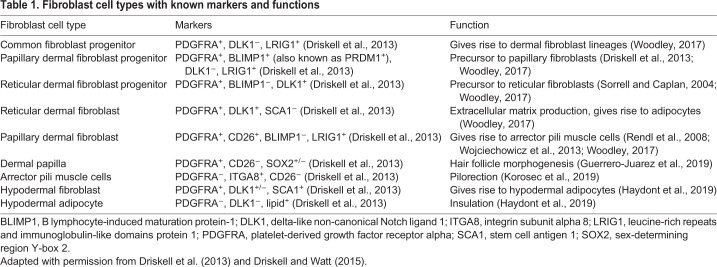
**Fibroblast cell types with known markers and functions**

Additionally, our group have identified four other distinct embryonic fibroblast lineages in the mouse dorsum: engrailed-1 (*En1*)-positive (*En1*^+^) and *En1*-negative (*En1^−^*) fibroblasts, and paired-related homeobox 1 (*Prrx1*)-positive (*Prrx1*^+^) and *Prrx1*-negative (*Prrx1*^−^) fibroblasts (Rinkevich et al., 2015). The fibroblasts marked by embryonic expression of *En1* are responsible for most of the dermal connective tissue deposition during cutaneous wound healing, radiation-induced fibrosis and cancer stroma (see Glossary, Box 1) in adult murine skin (Rinkevich et al., 2015). Furthermore, this fibrogenic fibroblast lineage could be identified through CD26 [also known as dipeptidyl peptidase 4 (DPP4)] expression, a finding with important translational implications because these markers could be used to specifically target the pro-fibrotic fibroblast subpopulation in humans (Rinkevich et al., 2015). Indeed, small molecule-based inhibition of DPP4 enzymatic activity during dorsal wound healing in mice significantly reduced scarring (Rinkevich et al., 2015). A follow-up study additionally demonstrated that dermal regeneration is driven by *En1*^–^ fibroblasts: the transition from scarring to regeneration can be reversed by transplanting *En1*^–^ cells into the dorsal dermis of recipient mice, highlighting that two fibroblastic lineages govern dermal development and the shift from regeneration to scarring (Jiang et al., 2018).

Embryonic expression of *Prrx1* can also be used to identify two distinct embryonic fibroblast lineages in the mouse ventral dermis: *Prrx1*^+^ and *Prrx1*^−^ fibroblasts (Hu et al., 2018). Analogous to *En1*^+^ fibroblasts in the dorsal dermis, *Prrx1*^+^ fibroblasts are the ventral lineage of scar-forming fibroblasts. *Prrx1*^+^ fibroblasts increase as a proportion of total fibroblasts within the ventral dermis over gestation, which corresponds to the transition from scarless to scarring wound repair, and are responsible for the majority of collagen production in the dermis following radiation, wounding and in tumor stroma formation (Hu et al., 2018). As previously shown in the dorsal dermis, ablation of *Prrx1*^+^ fibroblasts leads to decreased cutaneous scarring (Rinkevich et al., 2015).

Together, this evidence demonstrates that the embryonic origin of mouse fibroblasts determines their characteristics in the adult. However, the relevance of these findings needs to be studied in human skin to truly understand the clinical implications for wound healing and fibrosis. To our knowledge, there are no studies that have investigated the fibrotic response of human fibroblasts based on their embryonic origin.

One of the most intriguing concepts in skin biology is the anatomical regional specificity of fibroblasts. Even fibroblasts that share a common embryonic origin may exhibit heterogeneity based on their anatomical location and microenvironment (Rinn et al., 2006). It has long been known that fibroblasts from different anatomical sites have unique metabolic activities and interactions with epithelial cells in human and murine skin (Jahoda et al., 1984, 1993). For example, studies with human cells have shown that scalp dermal fibroblasts create long hairs if transplanted to the arm *in vivo*, which implies they retain a ‘memory’ of their position in the body and remain capable of activating that region-specific phenotype even when removed from their native niche (Jahoda et al., 1984).

The interrogation of fibroblast populations from different anatomical sites has also increased our understanding of the complexity of fibroblast heterogeneity. The ‘homeobox (HOX) code’ (see Glossary, Box 1) was proposed to dictate positional identity of the skin and influence site-specific epidermal differentiation (Rinn et al., 2008). Chang et al. (2002) interrogated this concept further by investigating the function of human skin fibroblasts based on their corresponding HOX gene expression profile. The authors used adult donor skin from ten different anatomical sites – including the arm, abdomen, back, scalp, foreskin, thigh, gum and toe – to establish fibroblast cultures (Chang et al., 2002). Transcriptomic analysis of these *in vitro* cultures revealed a striking relationship between fibroblast gene expression and site of origin, which was termed topographic differentiation. In another study, Rinn et al. (2006) hypothesized that dermal organization may arise due to the position on a coordinate system. They evaluated the genome-wide gene expression profiles of primary human fibroblasts from 43 unique anatomical sites spanning the human body, including the skin and internal organs (Rinn et al., 2006). Large differences in gene expression related to three primary anatomic divisions: (1) anterior versus posterior (rostral/caudal), (2) proximal versus distal, and (3) dermal versus non-dermal (Rinn et al., 2006). Genes involved in pattern formation, cell-cell signaling and matrix remodeling were differentially expressed, highlighting how fibroblast gene expression programs are related to their positional identities on the major anatomic axes.

To date, the extent of fibroblast heterogeneity based on anatomical location has only been studied in human skin and not in murine skin. The identification of the characteristic gene expression profiles of fibroblast subpopulations at specific anatomical sites in human and murine skin will allow researchers to develop targeted skin substitutes and to manipulate skin characteristics for cellular therapy applications. Further understanding into how HOX genes regulate dermal fibroblast behavior, and how these molecular findings translate into functional effects on fibroblast phenotype, is required to implement these findings for clinical translation.

## Fibroblast subpopulations in human and murine skin

Within human and murine skin, fibroblasts exhibit separation into functionally distinct subpopulations based on their location within the dermis itself. Indeed, the dermis has two distinct histological layers: papillary and reticular. Several studies have shown that the ECMs of these two subsites are different and that the fibroblasts isolated from both sites have different functional activities (Fig. 1, Table 1) (Ghetti et al., 2018; Harper and Grove, 1979; Wang et al., 2008). The long-known differences in the histological structure of papillary and reticular dermis has led the investigation into fibroblast heterogeneity within these two layers of the murine dermis (Driskell and Watt, 2015).

The papillary and reticular fibroblast subsets in murine skin are increasingly being defined based on their molecular markers (Fig. 3, Table 2). These two distinct lineages, which can be identified by immunostaining, also exhibit different functions (Driskell et al., 2013), as highlighted by transplantation of the two freshly isolated distinct fibroblast subpopulations into a silicone bubble-like chamber situated on the panniculus carnosus of an immunosuppressed mouse (Woodley, 2017). Transplantation of a mixed cell suspension results in the development of a tissue resembling skin with a typical dermal pattern, with both papillary and reticular compartments. If papillary fibroblasts are missing from the cell suspension, hair follicles and the papillary dermis fail to form. In contrast, absence of reticular fibroblasts produces skin lacking the reticular dermis and part of the hypodermis. Both fibroblast lineages also contribute differently to healing following full thickness excisional wounds on the adult mouse dorsum. Reticular fibroblasts migrate into the wounded site early, producing a collagen-rich dermis resembling a scar, and are incapable of regenerating hair follicles (Woodley, 2017). Conversely, papillary fibroblasts participate in the later phases of wound healing. The dominance of reticular fibroblasts in healing wounds may explain why *de novo* hair follicle formation rarely occurs upon wound healing (Woodley, 2017).

**Table 2.**
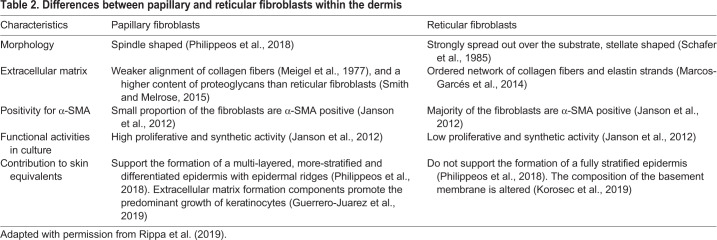
**Differences between papillary and reticular fibroblasts within the dermis**

Single-cell analysis can help to define the heterogeneity of poorly characterized cell types. Guerrero-Juarez et al. (2019) created very large unsplinted wounds (1.5 cm) on mouse dorsum and used this technique to reveal that wounding induces a high degree of heterogeneity among fibroblasts, showing that major populations of cells co-exist in the wound and revealing that some differentiate further towards myofibroblasts (see Glossary, Box 1), whereas others differentiate into non-myofibroblast lineages. Overall, the study found two main populations of fibroblasts 12 days after wounding on the basis of their transcription signatures and PDGFR expression patterns. The first, representing 24% of total wound fibroblasts, expressed low levels of transforming growth factor-beta (TGF-β; also known as TGFB1) receptors (TGF-βR2, TGF-βR3 and PDGFRA) and high levels of PDFGRB, and was comprised of three subclusters. The second group, the remaining 76% of total wound fibroblasts, showed high expression levels of TGF-βR2 and TGF-βR3, and high levels of PDGFRA, but not PDGFRB. PDGFRA and TGF-β signaling are known drivers of fibrosis in multiple tissues, including fat and skeletal muscle (Guerrero-Juarez et al., 2019). Examining the fibrotic potential of skin wound fibroblasts based on the variation of these markers may provide further understanding into human fibroblast heterogeneity. Another study analyzed human skin by single-cell RNA sequencing and found two major fibroblast populations with distinct expression of genes, including secreted frizzled-related protein 2 (*SFRP2*) and flavin-containing dimethylaniline monooxygenase1 (*FMO1*) (Tabib et al., 2018)*.* The two main populations, defined by expression of *SFPR2* and *FMO1*, had different morphologies and gene expression profiles, which suggest roles in matrix deposition and inflammatory cell retention (Tabib et al., 2018).

In murine skin, two additional fibroblast subpopulations have been identified in close association with hair follicle structures such as the hair follicle dermal papilla and the dermal sheath (Driskell et al., 2013). These fibroblasts are believed to coordinate the development, homeostasis and regeneration of epithelial structures, including the hair and epidermis, and show specific markers. However, their function in wounding has not been fully examined. The mouse dermis is also host to the pre-adipocyte, a fibroblast subtype that resides in the hypodermis or dermal white adipose tissue under the reticular dermis, and directly differentiates into adipocytes at postnatal day (P)2 (Fig. 3) (Driskell et al., 2013).

Although the work discussed above identified numerous surface markers for mouse fibroblasts, few studies focused on the human skin and accounted for reticular and papillary fibroblasts. A recently identified group of cell surface markers may help distinguish between the papillary and reticular fibroblasts of the human dermis (Korosec et al., 2019). Flow cytometry analysis of fibroblasts isolated from superficial and lower layers of the dermis showed that fibroblast activation protein-positive (FAP^+^) CD90^−^ (also known as THY1^–^) cells are enriched in the papillary dermis. Furthermore, papillary fibroblasts have increased proliferative potential, express podoplanin (*PDPN*) and netrin-1 (*NTN1*) and do not differentiate into adipocytes (Korosec et al., 2019). In contrast, FAP^+^CD90^+^ fibroblasts express high levels of actin alpha 2 (*ACTA2*), matrix glial protein (*MGP*), peroxisome proliferator-activated receptor gamma (*PPARG*) and CD36, and readily undergo adipogenic differentiation, a hallmark of reticular fibroblasts. The differences in the papillary and reticular fibroblast behavior observed parallel those of murine skin (Driskell et al., 2013). Another study has shown that human papillary and reticular fibroblast identity may also vary according to age, as shown by genome-wide profiling, which revealed that KN motif and ankyrin repeat domains 4 (*KANK4*), aggrecan (*ACAN*), pregnancy-specific beta 1-glycoprotein (*PSG1*) and collagen 11 alpha 1 (*COLXIA1*; also known as *COL11A1*) were upregulated in aged fibroblasts (Haydont et al., 2019).

In contrast, a recent study identified at least five functionally distinct human dermal fibroblast subpopulations, and papillary and reticular dermal cells had distinct gene expression profiles (Philippeos et al., 2018). The first fibroblast population was marked by CD90^+^CD39^+^ (also known as ENTPD1^+^) CD26^−^ as well as high expression of collagen chains such as collagen 6 alpha 5 (*COL6A5*), and primarily localized in the upper dermis. The second population defined by CD90^+^CD36^+^ surface expression, was abundant in the lower dermis and included pre-adipocytes. A third population had high expression of known pericyte markers, and two further populations were identified as CD90^+^CD39^+^CD26^+^ and Lin^–^CD90^+^CD39^–^ regulator of G protein signaling 5 (RGS5)^–^. The fibroblast subpopulation markers were rapidly lost in culture, despite the cells usually maintaining their fibroblast function, highlighting the need to work with cells freshly isolated from human skin. The response of cultured cells to interferon gamma (IFN-γ), however, was retained. The upper dermal fibroblasts demonstrated an anti-inflammatory phenotype in response to IFN-γ stimulation and were able to support epidermal reconstruction when introduced into a decellularized dermis (Philippeos et al., 2018). These findings highlight how *ex vivo* expansion of specific fibroblast subpopulations could herald the development of specific skin-based substitutes. The existing studies on the varied fibroblast cell surface markers in human unwounded skin underscore the extent of the future work needed to understand fibroblast heterogeneity in skin fibrosis.

## Regulation of fibroblast heterogeneity

Several signaling pathways have been implicated in the regulation of fibroblasts in fibrosis, the most studied among these being the Wnt pathway (Akhmetshina et al., 2012). Wnt signaling is a key regulator of embryonic development and organogenesis (Willert and Jones, 2006), and is involved in all phases of wound healing (Chen et al., 2017). Epidermal-dermal communication is maintained via the canonical Wnt signaling pathway, and activation of Wnt/β-catenin in mouse basal keratinocytes increases fibroblast proliferation and ECM formation (Chen et al., 2012). A deeper understanding of the relationship between epidermal cells and fibroblasts may provide clues to fibroblast heterogeneity. Hair follicle formation decreases with age and this effect depends on anatomical location (Rognoni et al., 2016). The loss of hair-forming ability in murine wounds was shown to be due to increased recruitment of reticular fibroblasts, which are unable to produce hair follicles and respond to Wnt/β-catenin signaling within the wound (Rognoni et al., 2016). However, more studies are needed to understand the precise role of the Wnt/β-catenin pathway within wounded human and murine skin.

Although it is clear that fibroblasts are under the control of regulatory signaling pathways, different fibroblast types also respond to distinct paracrine signals (Lichtenberger et al., 2016). Lichtenberger et al. (2016) examined how murine fibroblast subpopulations respond to epidermal Wnt activation. Upon Wnt/β-catenin activation, epidermal cells express Sonic hedgehog (Shh), which stimulates proliferation of and ECM remodeling by papillary dermal fibroblasts. In contrast, reticular dermal fibroblasts strongly respond to epidermal TGF-β signaling. To date, few studies have examined the regulatory control of fibroblast heterogeneity in both human and murine skin, which is crucial to improving the outcome of tissue fibrosis.

## Myofibroblast heterogeneity

One of the hallmarks of wound healing is wound contraction (see Glossary, Box 1) (Nedelec et al., 2000). The seminal work of the Gabbiani group established that tissue contraction is promoted by a specialized population of fibroblasts, the myofibroblasts (Desmouliere et al., 2004). Myofibroblasts are absent from normal tissue and become activated during wound healing (Plikus et al., 2017). Mature fibroblasts are cells with contractile properties, like smooth muscle cells, and express alpha-smooth muscle actin (α-SMA) (Desmouliere et al., 2004). However, α-SMA is not unique to this population and is also expressed by pericytes and endothelial cells (Morikawa et al., 2002). Myofibroblasts secrete collagen and form microfilament bundles, and are arguably the most important cells able to influence scarring, and fibrotic disease states are characterized by the progressive migration of abnormally high numbers of active myofibroblasts (Klingberg et al., 2013). Hence, identification of unique or enriched myofibroblast markers beyond α-SMA will aid the treatment of skin fibrosis by enabling myofibroblast-specific treatments.

Although originally considered a final differentiation state of fibroblasts, myofibroblasts are now recognized as a heterogeneous population of cells that derive from several progenitors. Specific subpopulations of dermal fibroblasts have greater myofibroblast potential (Lin et al., 2008). In animal models, PDGFRB^+^ pericytes and/or perivascular progenitor cells are the predominant myofibroblast source and can promote fibrosis in the skin, kidney and liver (Lin et al., 2008). Resident fibroblasts and bone marrow-derived cells also differentiate into myofibroblast lineages (Quante et al., 2011), but endothelial and epithelial cells do not (Humphreys et al., 2010). Thus, myofibroblasts can be generated from different sources.

An emerging concept in organ fibrosis is myofibroblast plasticity (Marangoni et al., 2015). Adipocytes can transdifferentiate to collagen-secreting myofibroblasts in the lung, liver and skin (Marangoni et al., 2015). Furthermore, epidermal injury stimulates hair follicle development and promotes the differentiation of human keloid-derived myofibroblasts into adipocytes through bone morphogenetic protein (BMP) signaling (Plikus et al., 2017). Recent evidence also shows that, during murine skin repair, macrophages activate the proliferation of a myofibroblast subpopulation called adipocyte precursors (APs), which have the capability for adipocyte lineage differentiation and dermal repair (Shook et al., 2018). AP proliferation is uniquely activated by CD301b (also known as MGL2)-expressing macrophages through platelet-derived growth factor-c (PDGFC) and insulin growth factor-1 (IGF-1) signaling (Shook et al., 2018). The observation that there are subsets of myofibroblasts within the wound bed that have precise interactions with immune cells could be the basis of future therapeutic developments in the fields of wound healing and fibrosis (Shook et al., 2018), although the role of myofibroblast heterogeneity in human skin fibrosis has not been evaluated to date.

## Importance of fibroblast heterogeneity for the clinical setting

Recent evidence has highlighted that fibroblast heterogeneity is particularly crucial during wound healing (Guerrero-Juarez et al., 2019). Identifying unique fibroblast cell populations that cause fibrosis has significant implications for disease diagnosis and treatment. The current available evidence shows that delivery of upper dermal fibroblasts to a wound could be important in resolving scar formation, since these cells do not typically contribute to early wound repair but could potentially promote regeneration. Although studies to date have focused on the role of papillary and reticular fibroblasts in wound repair, emerging evidence also highlights the vital role of myofibroblasts. Understanding how myofibroblast subpopulations function and signal, and how they are regulated by the microenvironment, will allow optimization of treatments under different pathological conditions. Furthermore, understanding the transition and crosstalk between fibroblasts, myofibroblasts and other (e.g. inflammatory) cell types will be of vital importance.

Many studies of fibroblast heterogeneity utilize *in vivo* models, in which splinted wounds are made on the mouse dorsum, which allows fibroblasts to be isolated following scar formation (Rinkevich et al., 2015). However, several other *in vitro* models of skin scarring exist (Box 2) (Lotz et al., 2017). Traditional fibroblast and keratinocyte monoculture and three-dimensional (3D) collagen gel assays are now being superseded by exciting skin-on-chip models, which can provide a better understanding of the cell-cell and cell-matrix environment that governs skin repair (Box 2) (Kwak et al., 2020). Future work should utilize skin-on-chip models to study fibroblast heterogeneity to advance our understanding of the extrinsic factors, and potentially the therapeutic interventions, that regulate skin fibrosis.

**Box 2. Examples of current models to examine skin repair**

**Excisional *in vivo* wound model:** the most common wound repair *in vivo* model is the excisional *in vivo* wound model. Splinted full thickness cutaneous wounds are usually created on the dorsum of the rodent. Histology and macroscopic analysis is then used to analyze the skin wounds, to study the fibroblast and keratinocyte response to skin wounding (Rinkevich et al., 2015).

**Hydrogel models:** fibroblasts and keratinocytes are embedded in collagen hydrogels, with keratinocytes seeded on top of the hydrogel to provide a 3D environment as opposed to a 2D environment. Hydrogel models can be used *in vitro* and *in vivo*. Such hydrogel systems are easy to produce and can mimic a dermo-epidermal equivalent. However, there can be problems with mechanical stability of the gel, introducing variability (Lotz et al., 2017; Maarof et al., 2019).

**Monolayer models:** fibroblasts and keratinocytes are grown in monolayers *in vitro*. This allows for the study of the cellular microenvironment of keratinocytes and fibroblasts individually. When studying keratinocytes, monolayers can form a differentiated epidermis. However, monolayer models do not assess the cellular interactions of skin or reconstitute the complex 3D environment of the cell-cell and cell-matrix interaction of the skin (Priestley and Lord, 1990; Wang et al., 2006).

**Skin-on-chip models:** skin-on-chip systems culture skin tissue within a microfluidic system, which can control many physical and biochemical parameters, including medium flow, mechanical force and biochemical gradients. These *in vitro* systems have the ability to study the 3D skin environment interactions to a greater depth than monolayer and hydrogel models but are very complex and require great expertise (Kwak et al., 2020; Lee et al., 2017).

Fibroblast heterogeneity could also hold promise for disease diagnosis as well as disease severity. For example, a study analyzing skin biopsies from 61 patients with scleroderma showed that expression profiles of their skin exhibited significant heterogeneity, and this was important for disease severity stratification and predicting the response to immunosuppressive treatments (Assassi et al., 2015). Understanding the precise role of distinct fibroblast subpopulations during fibrosis will allow treatment regimens to be optimized and thus provide better-targeted therapies in the future.

## Future work

Several groups have examined fibroblast heterogeneity in mouse embryonic mesenchymal cells using single-cell analysis and lineage tracing. Although there are numerous similarities between mouse and human skin, there are also a number of important differences. To date, most of the fibroblast subtypes have been identified in unwounded mouse skin; however, further work on unwounded human skin will show whether similar subtypes are conserved in human skin, and thus illuminate potential therapeutic avenues. The regulatory system underpinning fibroblast heterogeneity in human and murine skin remains unknown, with current studies focusing on the Wnt signaling pathway (Akhmetshina et al., 2012). Future work should aim to investigate the impact of other known fibrotic pathways on fibroblast heterogeneity in skin fibrosis.

## Conclusions

As discussed here, fibroblasts are not a uniform cell population. Their heterogeneity has been extensively studied in murine skin, but further work is needed in human skin. Emerging work has identified multiple subsets of myofibroblasts, the key cellular drivers of fibrosis; however, these specific populations and their distinct contributions to fibrosis have yet to be fully elucidated. Identifying the regulatory signals of specific fibroblast subpopulations will aid in the development of new therapies to prevent scarring and other fibroses and to improve wound repair. Thus, future studies unraveling the heterogeneity of human fibroblast subpopulations have the potential to unveil new directions for fibroblast cellular therapy within the field of regenerative medicine.
